# Daisaikoto improves fatty liver and obesity in melanocortin-4 receptor gene-deficient mice via the activation of brown adipose tissue

**DOI:** 10.1038/s41598-022-14371-y

**Published:** 2022-06-16

**Authors:** Shinichi Morita, Akira Sakamaki, Kyutaro Koyama, Osamu Shibata, Takashi Owaki, Chiyumi Oda, Atsushi Kimura, Taiki Nakaya, Katsuya Ohbuchi, Miwa Nahata, Naoki Fujitsuka, Norihiro Sakai, Hiroyuki Abe, Kenya Kamimura, Shuji Terai

**Affiliations:** 1 Division of Gastroenterology and Hepatology, Graduate School of Medical and Dental Sciences, Niigata University, 1-757, Asahimachi-dori, Chuo-ku, Niigata, 951-8510 Japan; 2 Tsumura Advanced Technology Research Laboratories, Tsumura & Co., 3586, Yoshiwara, Ami-machi, Inashiki-gun, Ibaraki, 300-1192 Japan; 3 Tsumura Kampo Research Laboratories, Tsumura & Co., 3586, Yoshiwara, Ami-machi, Inashiki-gun, Ibaraki, 300-1192 Japan; 4 Department of General Medicine, School of Medicine, Niigata University, 1-757, Asahimachi-dori, Chuo-ku, Niigata, 951-8510 Japan

**Keywords:** Metabolic diseases, Non-alcoholic fatty liver disease

## Abstract

Melanocortin 4 receptor gene-knockout (MC4R-KO) mice are known to develop obesity with a high-fat diet. Meanwhile, daisaikoto, one of Kampo medicines, is a drug that is expected to have therapeutic effects on obesity. Here, we report the efficacy of daisaikoto in MC4R-KO mice. Eight-week-old MC4R-KO male mice (n = 12) were divided into three groups as follows: the SD group, which is fed with a standard diet; the HFD group, fed a high-fat diet; and the DSK group, fed with a high-fat diet containing 10% of daisaikoto. After the four-week observation period, mice in each group were sacrificed and samples were collected. The body weights at 12 weeks were significantly higher in the HFD group than in the other groups, indicating that daisaikoto significantly reduced body weight gain and fat deposition of the liver. The metabolome analysis indicated that degradation of triglycerides and fatty acid oxidation in the liver were enhanced by daisaikoto administration. In MC4R-KO mice, the cytoplasm and uncoupling protein 1 expression of brown adipose tissue was decreased; however, it was reversed in the DSK group. In conclusion, daisaikoto has potentially improved fatty liver and obesity, making it a useful therapeutic agent for obesity and fatty liver.

## Introduction

The estimated global prevalence of non-alcoholic fatty liver disease (NAFLD) was 26.80% (95% confidence interval [CI] 23.47–30.42) in 2011–2015 according to a meta-analysis by Younossi et al.^1^, and was expected to increase now and in the future^2^. In addition, non-alcoholic steatohepatitis (NASH) has the potential to progress to liver fibrosis in the definition of NAFLD^3^. Liver fibrosis due to NASH can lead to poor prognosis by the progression of liver cirrhosis and hepatocellular carcinoma, similar to other etiologies of liver fibrosis. On the other hand, even patients with NAFLD have a significantly poorer prognosis than the general population, even to the malignancy of general organs and ischemic diseases^4^. However, medications to improve NAFLD has no standard; therefore, the European association for the study of the liver^5^ and the Japanese society of gastroenterology^6^ clinical guidelines for the management of NAFLD recommended weight loss by diet and/or exercise for therapy.

The melanocortin 4 receptor (MC4R) gene is mainly expressed in the feeding center of the hypothalamus and regulates food intake and energy expenditure^7^. The MC4R gene is also known to be a cause of hereditary obesity in humans^8^. In addition, MC4R gene-deficient mice with a high-fat diet have been known to be a NASH model that develops NASH-like fibrosis in 20 weeks and HCC in approximately a year based on the complications of obesity such as insulin resistance and dyslipidemia^9^. The MC4R gene also regulates the autonomic nervous system and energy production in brown adipose tissue in mice^10^; thus, the cause of obesity in MC4R gene knock out (MC4R-KO) mice is likely the feeding regulation. The MC4R gene is rarely expressed in the liver, and its deficiency is an excellent model of fatty liver and NASH for pathological analysis.

Daisaikoto is a herbal medicine that has been used for cholelithiasis, cholecystitis, jaundice, and liver dysfunction in Japan. Additionally, it has decreased the serum triglyceride levels^11^ and insulin resistance^12^ in animal studies, which indicated its potential therapeutic effects on fatty liver and obesity. Therefore, we examined the inhibitory effect of daisaikoto on obesity and NAFLD using MC4R-KO mice.

## Results

### Effect of daisaikoto on fat accumulation in MC4R-KO mice with a high-fat diet

**Figure 1:**
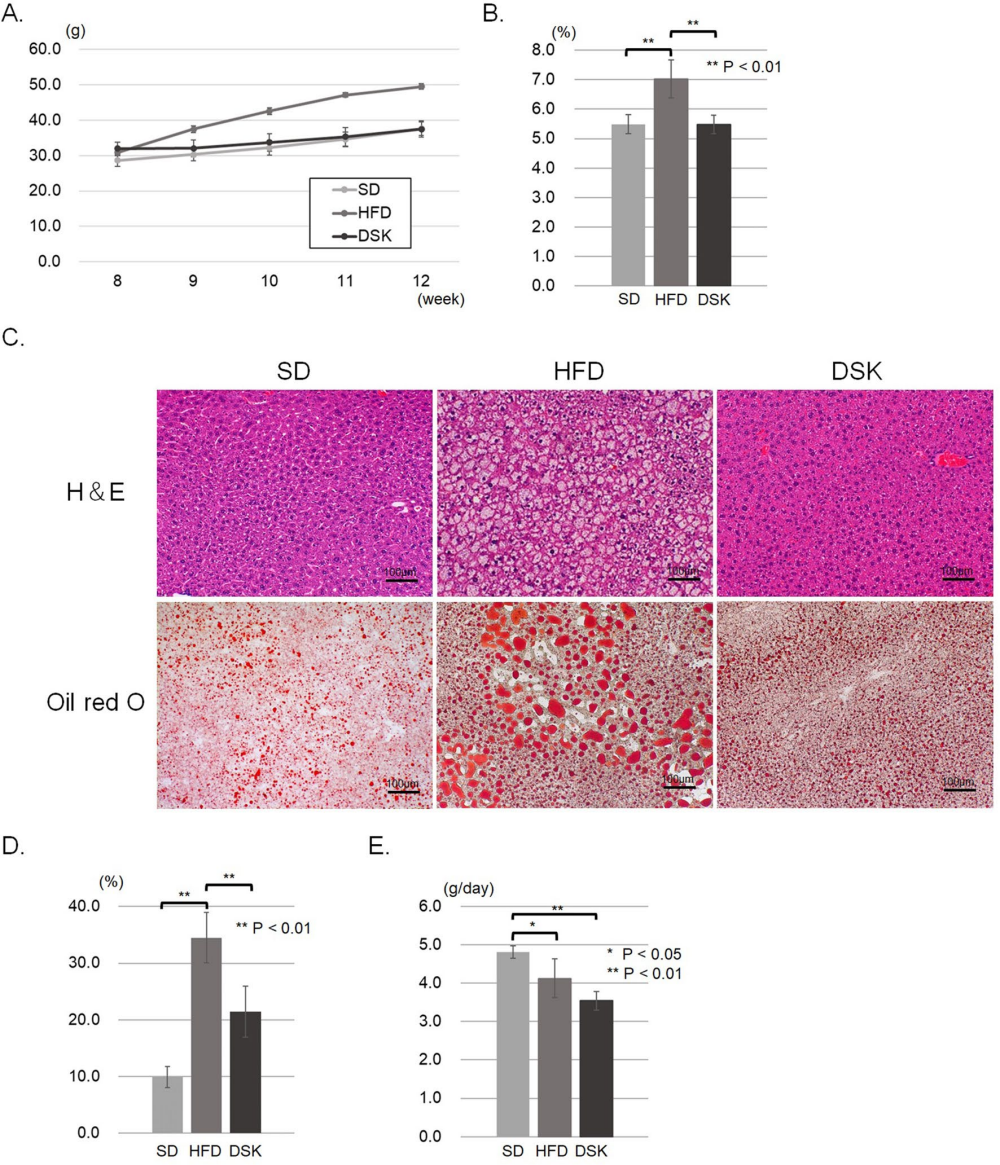
Effect of daisaikoto on fat accumulation in MC4R-KO mice with a high-fat diet. Changes in body weight during 4 weeks of every week (**A**, n = 4, P < 0.01). The liver-to-body weight ratio in the HFD group was significantly higher than in the other two groups (**B**, P < 0.01). H&E and oil red O staining indicated that fat deposition in the HFD group was significantly higher than that in the other two groups with a significant difference (**C,D**, P < 0.01). Meanwhile, no statistically significant differences were found in the dietary intake between the HFD and DSK groups (**E**). *MC4R-KO* melanocortin 4 receptor gene knock out, *H&E* hematoxylin and eosin.

The mean body weight of 8-week-old MC4R-KO mice was 28.7 ± 1.7 g, 30.9 ± 0.9 g, and 32.0 ± 1.7 g in the SD, HFD, and DSK groups, respectively. Figure 1A shows the body weight changes that were measured weekly from 8- to 12-week-old. The final body weights at 12 weeks of age were 37.6 ± 1.8 g, 49.5 ± 0.8 g, and 37.5 ± 2.4 g in the SD, HFD, and DSK groups, respectively, with a significant difference between these three groups (P < 0.01). The multiple comparison test revealed a significant difference between the SD and HFD groups (P < 0.01) and the DSK and HFD groups (P < 0.01) but no significant difference between the SD and DSK groups (P = 0.08). In addition, liver-to-body weight ratio at 12-week-old were 5.5% ± 0.3%, 7.0% ± 0.7%, and 5.5% ± 0.3% in the SD, HFD, and DSK groups, respectively. The ratio in the HFD group was significantly higher than in the other two groups (the SD and HFD groups, P < 0.01; the DSK and HFD groups, P < 0.01; Fig. 1B). Furthermore, the histological analysis revealed that the fat content area was 9.9% ± 1.9%, 34.5% ± 4.5%, and 24.1% ± 4.5% in the SD, HFD, DSK groups, respectively, with a significant difference (P < 0.01, Fig. 1C,D).

On the other hand, in the wild mice, the high fat diet group was also significantly higher than the standard diet or daisaikoto groups (the body weights at 12 weeks of age were 24.3 ± 0.5 g, 30.0 ± 1.0 g, and 23.6 ± 1.7 g in the WT-SD, WT-HFD, and WT-DSK groups, respectively, P < 0.01, supplemental Fig. 1A), without the fat accumulation in the liver (liver weight to body weight ratio at 12-week-old were 4.8 ± 0.1%, 4.3 ± 0.2%, and 4.4 ± 0.5% in the WT-SD, WT-HFD, and WT-DSK groups, respectively, P = 0.24; supplemental Fig. 1B).

Dietary intake was significantly higher in the SD group than in the other two groups; however, no significant differences were found between the HFD and DSK groups (4.8 ± 0.2 g/day, 4.1 ± 0.5 g/day, and 3.5 ± 0.2 g/day in the SD, HFD, and DSK groups, respectively; between the DSK and HFD groups, P = 0.09; Fig. 1E). In the wild type mice, no significant differences were found between these three groups (P = 0.25; supplemental Fig. 1C).

These results indicated that the fat deposition of the liver was observed in four weeks of high fat diet loading in MC4R-KO mice, and daisaikoto reduced the body weight gain and the fat deposition of the liver without decreasing food intake in MC4R-KO mice.

### Effect of daisaikoto on fat metabolism in the liver

**Figure 2:**
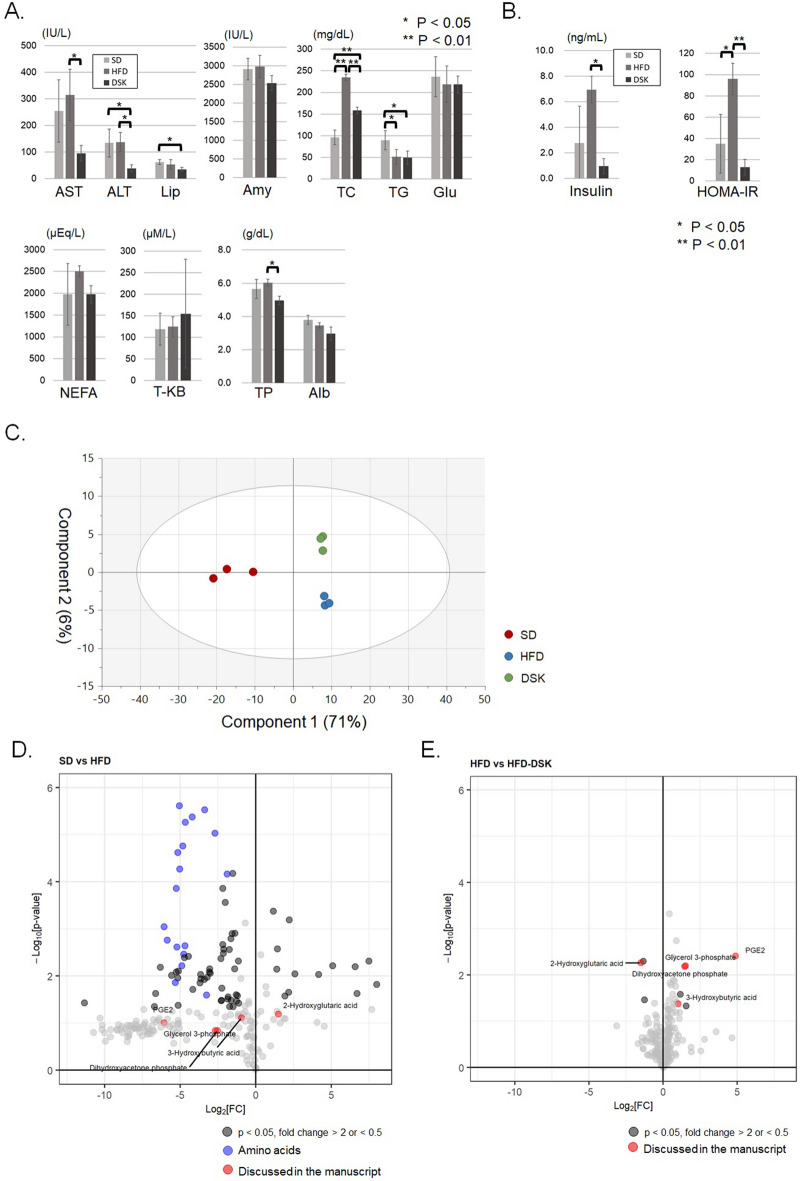
Effect of daisaikoto on fat metabolism in the liver. Total cholesterol and total protein levels were significantly lower in the DSK group than that in the HFD group; however, lipase, TG, glucose, and blood ketone levels were not significantly different (**A**). Serum insulin levels and HOMA-IR were significantly lower in the DSK group than that in the HFD group (**B**, p < 0.05 and p < 0.01, respectively). The PLS-DA score plot showed that component 1 clearly (71%) discriminated against the effect of HFD and component 2 contributed to the separation of the DSK and HFD groups albeit at a low contribution (**C**). A volcano plot demonstrates that many amino acid levels in the liver were significantly decreased during HFD treatment (**D**, P < 0.05, fold change > 2 or < 0.5). Regarding the effect of daisaikoto, the volcano plot indicated that daisaikoto administration changed 9 metabolite levels in the liver compared to the HFD group (**E**). *AST* aspartate aminotransferase, *ALT* alanine aminotransferase, *Lip* lipase, *Amy* amylase, *TC* total cholesterol, *TG* triglyceride, *Glu* glucose, *NEFA* non-esterified fatty acid, *T-KB* total ketone body, *TP* total protein, *Alb* albumin, *HOMA-IR* homeostasis model assessment-estimated insulin resistance, *PLS-DA* partial least squares-discriminant analysis.

Blood biochemical analysis showed that liver deviation enzymes, total cholesterol, and total protein levels were significantly lower in the DSK group than in the HFD group, but lipase, triglyceride, glucose, and blood ketone levels were not significantly different (Fig. 2A). In addition, serum insulin levels and homeostasis model assessment-estimated insulin resistance (HOMA-IR) were significantly lower in the DSK group than in the HFD group (Serum insulin levels were 2.8 ± 2.9 ng/mL, 7.0 ± 1.1 ng/mL, and 1.0 ± 0.6 ng/mL in the SD, HFD, and DSK groups, respectively, p < 0.05; HOMA-IR were 34.9 ± 27.6, 96.0 ± 14.8, and 13.0 ± 7.4 in the SD, HFD, and DSK groups, respectively, p < 0.01; Fig. 2B). These results indicate that daisaikoto can improve liver injury, cholesterol metabolism, and glucose intolerance due to fat deposition in the liver.

Supplemental Table 1 shows the list of evaluated metabolites and results by liver metabolome analysis. The partial least squares-discriminant analysis (PLS-DA) score plot showed that component 1 clearly (71%) discriminated against the effect of HFD (Fig. 2C). Component 2 contributed to the separation of the DSK and HFD groups, albeit at a low contribution (6%). The volcano plot, which arranged the metabolites along the axes of biological and statistical significance (p < 0.05, fold change > 2 or < 0.5), demonstrated that many amino acid levels in the liver were significantly decreased in the HFD treatment (Fig. 2D).

Regarding the effect of daisaikoto, a volcano plot indicated that daisaikoto administration changed 9 metabolite levels in the liver compared to the HFD group (Fig. 2E and supplemental Fig. 2). Daisaikoto treatment significantly increased the liver glycerol-3-phosphate and dihydroxyacetone phosphate, the degradation products of triglyceride, and 3-hydroxybutyric acid, which is a ketone metabolized from free fatty acids, and pantothenic acid and carnitine that is associated with the metabolism of fatty acids. These results would indicate the enhancement of triglyceride degradation and fatty acid oxidation in the liver. Furthermore, prostaglandin E2 (PGE2), which is a pro-inflammatory mediator, was also increased with daisaikoto administration. 2-hydroxyglutaric acid was increased in the HFD group and significantly decreased in the DSK group.

### Effect of daisaikoto on fat absorption

**Figure 3:**
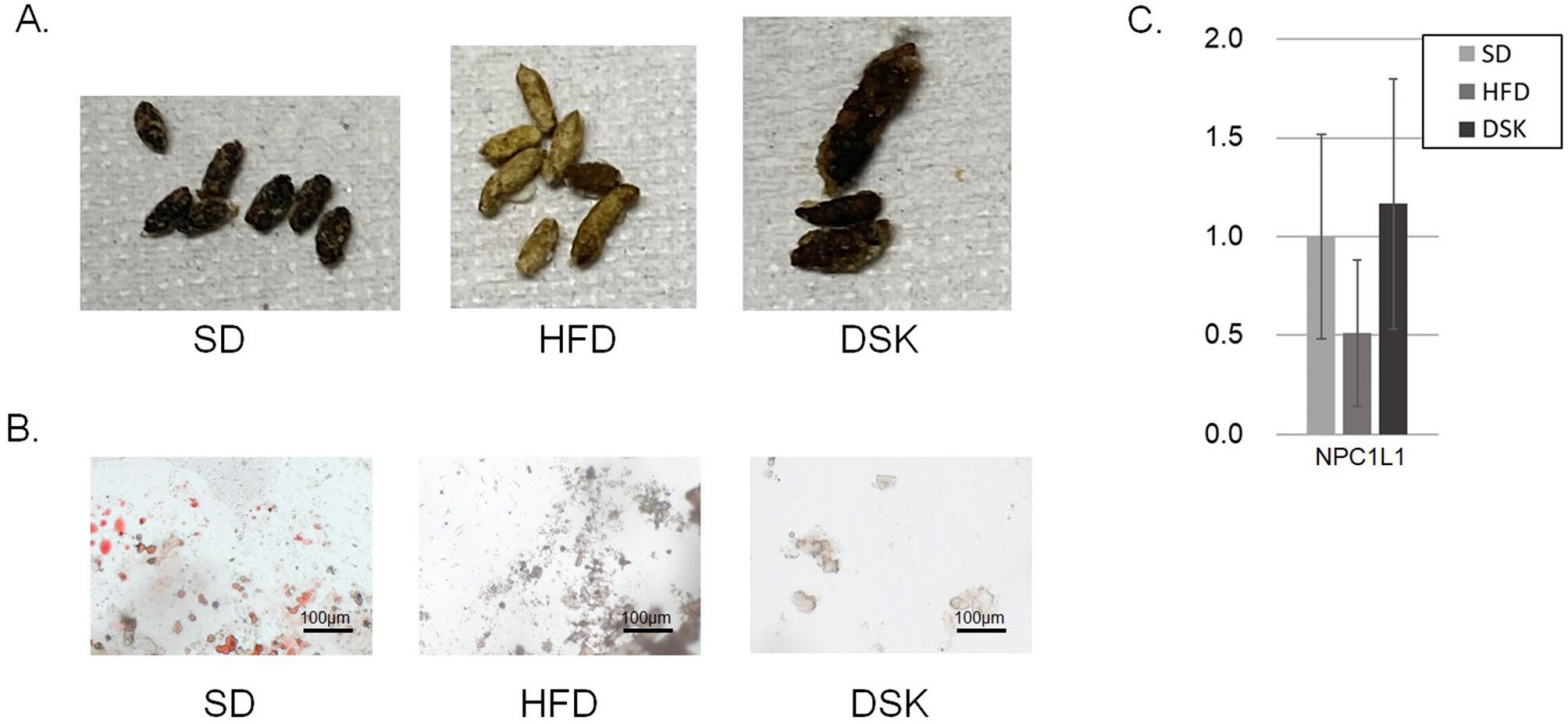
Effect of daisaikoto on fat absorption. The gross findings of feces (**A**) and oil red O staining (**B**) indicated no fatty stools in any of the three groups. The expression of NPC1L1 in the small intestine was evaluated by real-time PCR, which revealed no significant changes between the three groups (**C**). *NPC1L1* Niemann-pick C1 like 1.

Figure 3A shows the gross findings and 3B indicated the oil red O staining of the feces. No fatty stools were observed in any of the three groups. In addition, the expression of Niemann-pick C1 Like 1 (NPC1L1) in the small intestine was evaluated by real-time PCR, and no significant changes were observed among the three groups (Fig. 3C). Therefore, daisaikoto might have minimal effects on fat absorption of the gastrointestinal tract in MC4R-KO mice.

### Effect of daisaikoto on energy expenditure via brown adipose tissue

**Figure 4:**
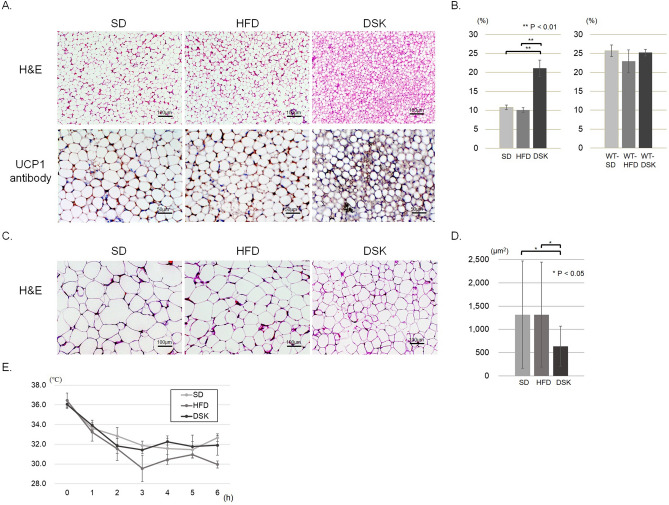
Effect of daisaikoto on energy expenditure via brown adipose tissue. The area of cytoplasm and UCP1 expression in MC4R-KO mice were significantly lower than those in the wild type mice, and that in the DSK group was significantly higher than those in the SD and HFD groups (**A,B**, P < 0.01). In addition, size measurement of the cross-sectional area of white adipocytes in the visceral fat showed a significant reduction in the DSK group compared with the HFD group (**C,D**, P < 0.01). Furthermore, the cold tolerance test indicated that the body temperature of the HFD group was lower than that of the other two groups after 6 h of cold stimulation of 4 °C (**E**, P < 0.01). *UCP1* uncoupling protein 1.

Hematoxylin and eosin (H&E) staining of the brown adipose tissue indicated that brown adipocytes had been whitened in MC4R-KO mice with and without a high-fat diet (SD and HFD groups) compared to wild type mice (supplemental Fig. 1D); however, the brown adipocytes in the DSK group remained in the cytoplasm. Immunohistochemistry by uncoupling protein 1 (UCP1) antibody indicated that the area of UCP1 expression was not affected by high fat diet or daisaikoto administration in wild type mice (25.7 ± 1.5%, 23.0 ± 3.0%, and 25.2 ± 0.9% of the WT-SD, WT-HFD, and WT-DSK groups, respectively; Fig. 4B and supplemental Fig. 1D). In MC4R-KO mice, the area of UCP1 expression significantly lower than those in the wild type mice (P < 0.01), and that in the DSK group was significantly higher than that in the SD and HFD groups (10.8% ± 0.6%, 10.1 ± 0.6%, and 21.1 ± 2.1% of the SD, HFD, and DSK groups, respectively, P < 0.01, Fig. 4A,B). In addition, the size measurement of the cross-sectional area of white adipocytes in the visceral fat revealed a significant reduction in the DSK group compared with the HFD group (1320 ± 1150 µm^2^, 1320 ± 1130 µm^2^, 640 ± 430 µm^2^ in the SD, HFD, and DSK groups, respectively, P < 0.01, Fig. 4C,D). These results suggest that daisaikoto improved heat production due to the reversed function of brown adipose tissue with UCP1 expression, which was inhibited by the MC4R gene insufficiency.

Furthermore, the thermogenic potential of the mice by the cold tolerance test indicated that the body temperature of the HFD group was lower than that of the other two groups after 6 h of cold stimulation of 4 °C (32.7 °C ± 0.4 °C, 30.0 °C ± 0.4 °C, and 31.9 °C ± 1.0 °C in the SD, HFD, and DSK groups, respectively, P < 0.01, Fig. 4E). These results suggest that daisaikoto improved the cold tolerance in the MC4R-KO mice with a high-fat diet.

## Discussion

Only 60% of obesity is suppressed in MC4R generally deficient mice using a modified Cre-loxP system after the MC4R expression is restored to the paraventricular nucleus of the hypothalamus and amygdala neurons^13^. In these mice, the overeating observed in MC4R general deficient mice is completely suppressed but energy metabolism was not improved. It has been suggested that sympathetic nerve activity from the lateral hypothalamus is one of the factors involved in energy metabolism in MC4R-KO mice.　The restored MC4R expression of the lateral hypothalamus in MC4R generally deficient mice induced the increased UCP1 expression in the intrascapular brown adipose tissue via the sympathetic nerve activation^10^. In addition, brown and white adipose are reversible in brown adipose tissue^14^, and vitamin E agonists^15^, β3-adrenergic agonists^16^, peroxisome proliferator-activated receptor γ agonists^17^, and cold exposure^18^ are known to be involved in browning. In this study, we revealed that MC4R-KO mice showed the reduction of the UCP1 expression in brown adipose tissue and increased cell diameter of visceral adipocytes, independent of the high-fat loading, which indicate sympathetic-mediated adipocyte dysfunction was developed as was done in previous studies^10^. Therefore, daisaikoto was thought to exert anti-obesity effects in MC4R gene-deficient mice by recovering of the homeostatic heat production and basal metabolism inhibited by MC4R deficiency.

Brown adipose tissue has been identified as a site of non-behavioral heat production in small rodents such as mice and hamsters and contributes to the rise and maintenance of body temperature in cold environments or upon awakening from hibernation. In detail, the information of physiological stimulation due to cold exposure is transmitted to the brain via transient receptor potential channels, and the information triggers various responses in the somatic and autonomic nervous systems^19^. One of the responses is the sympathetic nerve activation that is densely distributed in brown adipose tissue. By activating the sympathetic nerves, noradrenaline is released and acts on beta-adrenergic receptors in the brown adipose tissue, which induces the resolution of intracellular triglycerides. The released fatty acids serve as a substrate for heat production and directly activate the UCP1, a specific mitochondrial heat-producing protein, which induces heat production^20^. Furthermore, noradrenaline also acts on white adipose tissue and induces lipolysis by a similar mechanism. The fatty acids that are produced in white adipose tissue are released into the blood and consumed in the muscle or brown adipose tissue^21^. In this study, serum triglyceride and free fatty acid levels were not altered by the administration of daisaikoto. In MC4R-KO mice, triglycerides were accumulated in adipocytes and hepatocytes and were thought to raise a small amount in the blood.

Liver metabolomics revealed that daisaikoto affects energy metabolism, especially induced lipolysis and fatty acid oxidation, which might be involved in reduced fat deposition. In addition, PGE2 was also increased by the daisaikoto administration. PGE2 was known as a pro-inflammatory mediator; however, PGE2 attenuated fat deposition in mouse primary hepatocytes^22^. Furthermore, 2-Hydroxyglutaric acid, which was reported to be oncometabolite^23^, was increased in the HFD group and significantly decreased in the DSK group. Furhter experiments should be performed to confirm the mechanism, but these results suggest that dasaikoto may reduce lipotoxicity with a high-fat diet.

Several reports indicated the anti-obesity effect of daisaikoto; however, its mechanism has not been confirmed. The inhibition of the activity of lipase and lipid absorption in the intestines^11^, the promotion of the glucose uptake into adipocytes^24^, and the modulation of the gut microbiota^25^ are previously reported as mechanisms of the anti-obesity effect of daisaikoto. Meanwhile, no previous reports were found about the relationship between daisaikoto and the function of brown adipose tissue in our literature survey. In the study, daisaikoto improved the function of brown adipose tissue, heat production, and energy expenditure, which promoted lipolysis in the liver and improved insulin resistance. This mechanism was not observed in the wild type mice, in which brown adipose tissue function is preserved. Moreover, fat absorption was hardly inhibited in this study. In MC4R-KO mice, the increased energy expenditure may be a primary cause of the anti-obesity effect; therefore, it exceeded the effect of the inhabitation of lipid absorption. In addition, the actual active ingredients and their direct mechanisms have not been elucidated because daisaikoto is a combination of many herbal medicines. Previous reports have shown that scutellariae radix^11^ and its component, baicalin^24^, have the main beneficial ingredients; however, other ingredients may also have supplementary or combined anti-obesity effects.

In conclusion, daisaikoto showed anti-obesity effects by salvaging the dysfunction of brown adipose tissue in MC4R-KO mice. Daisaikoto is a combination of various crude drugs, thus several mechanisms contribute to the anti-obesity effects, including an increased energy expenditure. Daisaikoto has the potential to improve fatty liver and obesity, making it a useful therapeutic agent for obesity and NAFLD.

## Methods

### Animals

MC4R-KO mice with a C57BL/6J background were kindly provided by Dr. Takayoshi Suganami (Nagoya University) and Dr. Yoshihiro Ogawa (Kyushu University). Eight-week-old C57BL/6J wild male mice were purchased from Charles River (Yokohama, Japan). Four randomly selected mice were housed in individual cages under controlled environmental conditions (temperature of 20–23 °C, humidity of 45–55%, 12-h dark/light cycles), and the animals were allowed free access to food and water in specific pathogen-free facilities. All animal experiments were conducted in compliance with the guidelines which were reviewed by the Institutional Animal Care and Use Committee of Niigata University and this study is approved by the President of Niigata University (approval number: SA00543). The study was carried out in compliance with the ARRIVE guidelines.

### Development of animal models

All MC4R-KO male mice (n = 12) were fed CE-2 (CLEA Japan, Inc., Tokyo, Japan), the standard diet for mice, until the age of 8 weeks. The mice were randomly divided into three groups as follows: the SD group, HFD group, and DSK group. Wild type mice (n = 12) were also randomly divided into three groups as follows: the WT-SD group, WT-HFD group, and WT-DSK group. The SD and WT-SD groups mice continued receiving CE-2. The HFD and WT-HFD groups were fed 90% of a high-fat diet (Western Diet D12079BM, including 41 kcal% of fat, Research Diets, Inc., New Brunswick, NJ, USA) with 10% of CE-2. The DSK and WT-DSK groups were fed the 90% of Western Diet containing 10% of daisaikoto (Tsumura and Co., Tokyo, Japan). After the four-week observation, at the age of 12 weeks, mice in each group were sacrificed by cervical dislocation, and blood was collected from the heart immediately. Liver tissues, brown adipose tissue from the intrascapular area, and white adipose tissue from visceral fat were removed, and then portions of the samples were stored in 10% formalin solution. Other portions of the liver and feces that are collected in the colon were embedded in optimal cutting temperature compounds and frozen and stored at − 80 °C. Feces, intestines, and the remaining samples of the liver were immediately frozen in liquid nitrogen and stored at − 80 °C.

### Histological analysis

Liver and adipose tissue samples were fixed in 10% formalin before being embedded in paraffin and stained with H&E and immunohistochemistry. Immunohistochemistry used UCP1 antibody (GTX112784; GeneTex, Inc., CA, USA) at 1:500 dilution with Vectastain Elite ABC rabbit IgG kit (PK-6101; Vector Laboratories, CA, USA), and DAB chromogen tablets (Muto Pure Chemicals, Tokyo, Japan).

Oil red O (ORO) staining was performed using frozen sections that are embedded in an optimal cutting temperature compound. The ORO working solution was prepared following a previously published protocol^26^. Frozen sections were cut into 10-µm slices by cryostat and dried at room temperature for 10 min. Next, the ORO working solution was added to cover the sections and incubated at room temperature for 5 min. The sections were rinsed under running tap water for 30 min. The slides were mounted with a water-soluble mounting medium and covered with coverslips.

Images were captured for each tissue section randomly and quantitatively analyzed on ImageJ software (version 1.8.0_172; National Institutes of Health, Bethesda, MD, USA) with an RGB-based protocol, as previously reported^27^.

### Blood chemistry and cholesterol concentrations

Serum levels of alanine aminotransferase, aspartate transaminase, lipase, amylase, total cholesterol, triglyceride, glucose, non-esterified fatty acid, total ketone bodies, total protein, and albumin were determined by Oriental Yeast Co., LTD. Nagahama LSL (Nagahama, Japan). Serum samples were applied for an insulin enzyme-linked immunosorbent assay kit (M1104; Morinaga Institute of Biological Science, Inc., Yokohama, Japan) according to the manufacturer’s instructions. Insulin resistance was evaluated according to the HOMA-IR index.

### Liver metabolomics

Hepatic hydrophilic metabolites and lipid mediators were measured by liquid chromatography-tandem mass spectrometry and gas chromatography-tandem mass spectrometry. Analyses were conducted using the Method Packages (Shimadzu, Kyoto, Japan), which contains a mass spectral library, method files specifying the analytical conditions, and data analysis parameters. Liver metabolomics and the analysis of the results were provided by Tsumura & Co.

### Expression of NPC1L1 in the small intestine

The total RNA was extracted from the small intestine using an RNeasy Mini kit (Qiagen, Hilden, Germany) and was reverse-transcribed into cDNA using a QuantiTect Reverse Transcription kit (Qiagen). The expression of NPC1L1 protein and glyceraldehyde 3-phosphate dehydrogenase (Gapdh) was quantified using quantitative PCR with SYBR Green and the StepOnePlus System (Thermo Fisher Scientific, Waltham, MA, USA), and the results were analyzed with the bundled software. The following primers were used: NPC1L1 (forward), 5’-ATCCTCATCCTGGGCTTTGC-3’; NPC1L1 (reverse), 5’- GCAAGGTGATCAGGAGGTTGA-3’; and Gapdh (QT01658692; Qiagen). Changes in gene expression were evaluated using the 2^−ΔΔCt^ method, with normalized gene expression to that of Gapdh in each sample.

### Cold tolerance test

The rectal temperature of 12-week-old mice in each group was measured using a thermometer (AD-1687, A&D Company, limited. Tokyo, Japan). The rectal temperature was measured at room temperature as a baseline, then the cages were moved to a cold room at 4 °C, and the body temperature was measured six times every hour.

### Statistical analysis

Continuous data were presented as means ± standard deviation while categorical data were presented as frequencies and percentages. The groups were compared using the one-way analysis of variance (ANOVA) or the two-way repeated-measures ANOVA with the Tukey–Kramer method. The threshold was set at P < 0.05 for statistical significance. The calculations were performed using GraphPad Prism ver. 6.0. Metabolomics data were processed using R software (R Foundation, Vienna, Austria). In addition, R was also used to calculate the statistical significance of the differences between two points using Welch’s *t*-test. PLS-DA was performed using SIMCA 17 (Umetrics, Umeå, Sweden).

## Supplementary Information

Supplementary Figure 1.

Supplementary Figure 2.

Supplementary Table 1.
