# Supplementary figures and images for: Daisaikoto improves fatty liver and obesity in melanocortin-4 receptor gene-deficient mice via the activation of brown adipose tissue
Source: Sci Rep. 2022 Jun 16;12:10105. doi: 10.1038/s41598-022-14371-y (PMC9203505; doi:10.1038/s41598-022-14371-y)

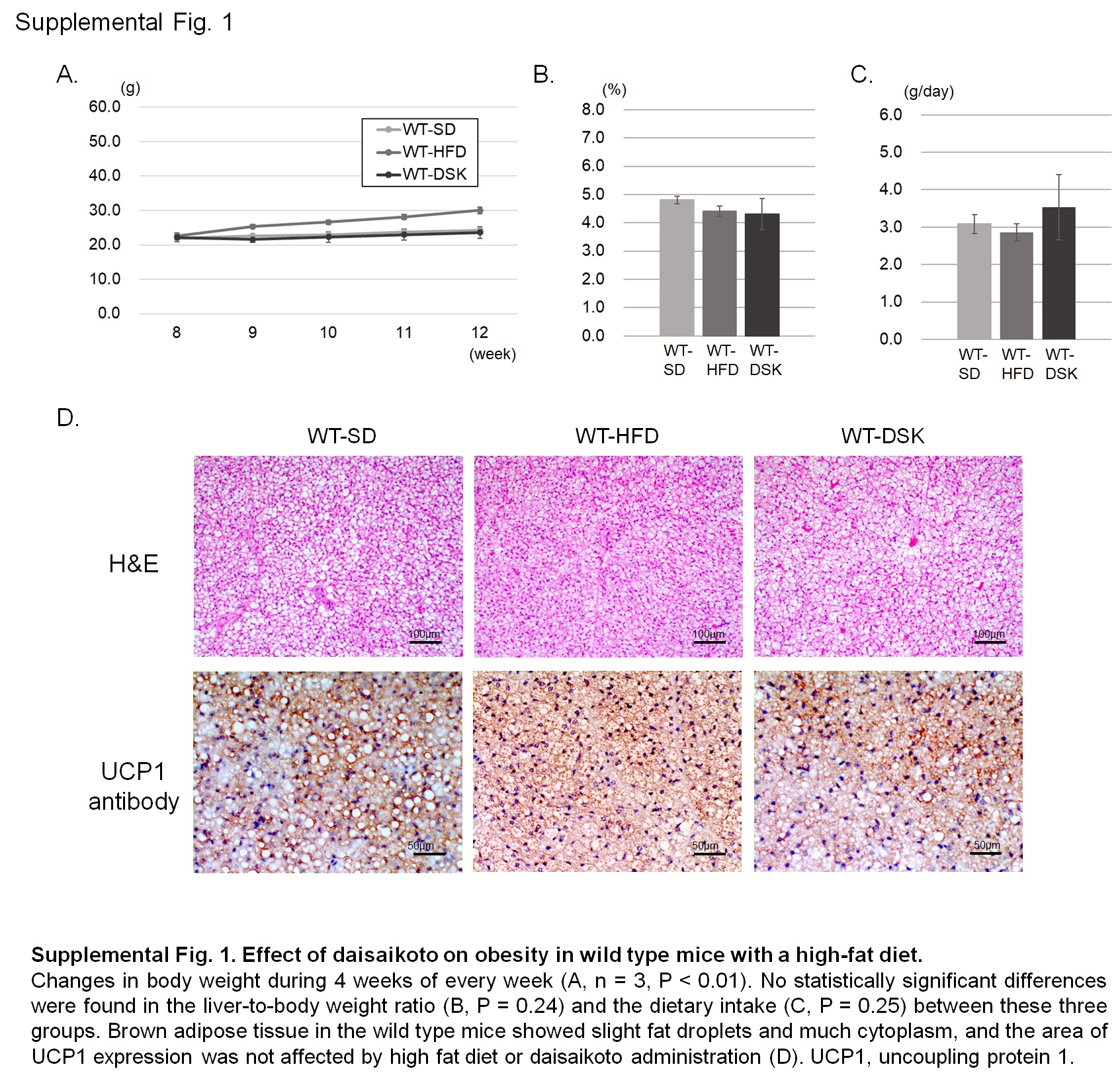

Supplement: Supplementary file 1 — Supplementary Figure 1. [file 41598_2022_14371_MOESM1_ESM.jpg]

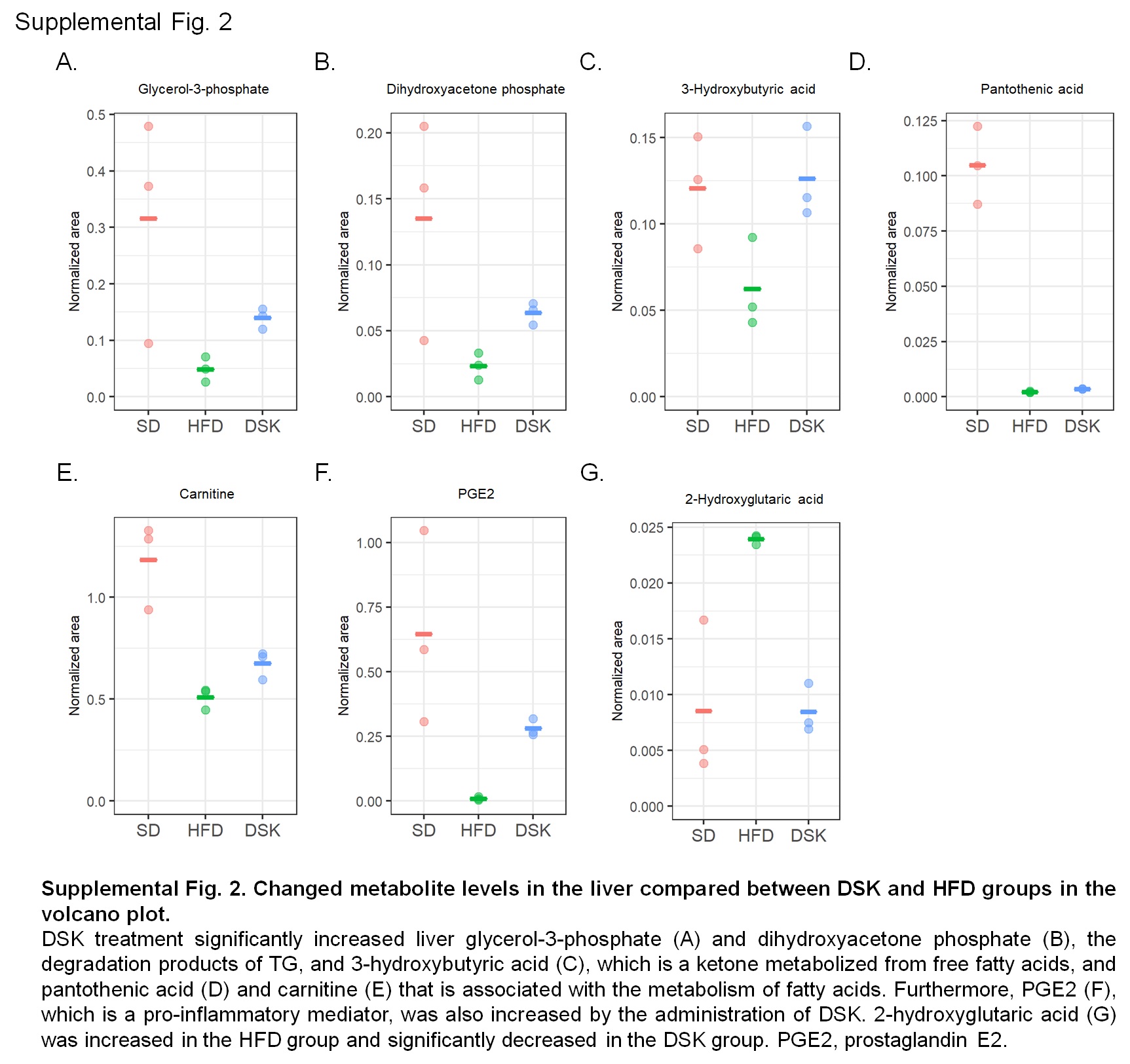

Supplement: Supplementary file 2 — Supplementary Figure 2. [file 41598_2022_14371_MOESM2_ESM.jpg]
